# Post-Transcriptional Regulation of Hepatic DDAH1 with TNF Blockade Leads to Improved eNOS Function and Reduced Portal Pressure In Cirrhotic Rats

**DOI:** 10.1038/s41598-017-18094-3

**Published:** 2017-12-20

**Authors:** V. Balasubramanian, G. Mehta, H. Jones, V. Sharma, N. A. Davies, R. Jalan, R. P. Mookerjee

**Affiliations:** Liver Failure Group, Institute for Liver and Digestive Health, University College London, London, UK

## Abstract

Portal hypertension (PH) is a major cause of morbidity and mortality in chronic liver disease. Infection and inflammation play a role in potentiating PH and pro-inflammatory cytokines, including TNF, are associated with severity of PH. In this study, cirrhotic bile duct ligated (BDL) rats with PH were treated with Infliximab (IFX, a monoclonal antibody against TNF) and its impact on modulation of vascular tone was assessed. BDL rats had increased TNF and NFkB compared to sham operated rats, and their reduction by IFX was associated with a reduction in portal pressure. IFX treatment also reduced hepatic oxidative stress, and biochemical markers of hepatic inflammation and injury. IFX treatment was associated with an improvement in eNOS activity and increased l-arginine/ADMA ratio and DDAH1 expression. *In vitro* analysis of HepG2 hepatocytes showed that DDAH1 protein expression is reduced by oxidative stress, and this is in part mediated by post-transcriptional regulation by the 3′UTR. This study supports a role for the DDAH1/ADMA axis on the effect of inflammation and oxidative stress in PH and provides insight for new therapies.

## Introduction

Portal hypertension (PH) is a serious complication of chronic liver disease and a major cause of morbidity and mortality. It is associated with the development of other complications, such as variceal haemorrhage, hepatic encephalopathy, and ascites, and is recognised as a key prognostic step in the progression of cirrhosis. PH arises from increased hepatic resistance to blood flow. The pathobiology involves both alterations in liver tissue architecture, with the development of fibrosis, and endothelial dysfunction, which causes increased intrahepatic vascular tone^1,2^. In addition, infection and inflammation are recognised as playing a role in further potentiating PH and complications of cirrhosis.

Bacterial translocation, measured by its surrogate marker serum bacterial DNA, is correlated with both systemic inflammation and PH^3^. The pro-inflammatory cytokines, TNF and IL-6, are elevated in cirrhosis patients and increase further in the context of infection. This inflammatory response is associated with severity of PH and its complications^4^. Indeed, TNF levels were found to be higher in alcoholic hepatitis patients who subsequently died than in those who survived^5^.

Infliximab (IFX) is a recombinant chimeric monoclonal antibody against TNF. It has a high affinity for both soluble and membrane bound TNF, and prevents TNF binding to its receptors resulting in neutralisation of the biological activity of TNF^6–8^. IFX has been used therapeutically for several immune mediated disorders that involve elevated TNF including inflammatory bowel disease, rheumatoid arthritis, and ankylosing spondylitis^9–13^. Early clinical trials using IFX in alcoholic hepatitis patients showed promising results^14–16^ with improvements in Maddrey’s discriminant function score, inflammatory indices, and systemic and hepatic haemodynamics. However, a subsequent study using a combination of high doses of prednisolone and IFX was halted due to adverse outcomes^17,18^. Despite significant differences in dose and study design^17,19^ controversy remains around the applicability of IFX treatment in alcoholic hepatitis^20,21^. Many investigators^14–17,19,22^ concluded that there is a need for large randomised trials of IFX in AH, although this has not been achieved to date, possibly due to the controversies surrounding earlier studies. A greater understanding of the mechanisms by which IFX promotes potential beneficial effects in cirrhosis and haemodynamics would facilitate its use in cirrhosis to be revisited, and importatntly, would lead to the development of other related therapies.

Nitric oxide (NO), produced from l-arginine by nitric oxide synthase (NOS), has a broad range of biological functions. In the liver, NO produced by the endothelial NOS isoform (eNOS) is a key regulator of intrahepatic vascular tone^23^. Altered metabolism or release of NO has been implicated in various vascular and inflammatory pathologies^24,25^. NO can also be produced by the inducible NOS (iNOS) isoenzyme. Expression and activity of iNOS are induced by sepsis and inflammation. This inappropriate, induced NO production leads to the generation of the free-radical peroxy-nitrite. Peroxy-nitrite, in turn, causes oxidative stress resulting in lipid peroxidation and the production of protein adducts of peroxidised lipids such as 4-hydroxynonenol (4-HNE).

Decreased eNOS activity has been implicated in the pathobiology of portal hypertension. This is despite normal, or even increased, levels of hepatic eNOS protein expression in cirrhosis^26–28^. This apparent paradox suggests that post-translational modification or the presence of eNOS inhibitors may be the cause of reduced eNOS activity. Asymmetric dimethyl arginine (ADMA) is an endogenous inhibitor of eNOS^29^. We and others have previously shown that ADMA levels are elevated in cirrhotic patients, and correlate with both severity of PH and systemic inflammatory response syndrome (SIRS)^30–32^. ADMA is metabolised by dimethylarginine dimethylaminohydrolase (DDAH), with the DDAH1 being the isoenzyme primarily responsible for ADMA breakdown^33,34^. Hepatic DDAH1 expression is reduced in cirrhosis patients and rat models^35^. DDAH1 activity is sensitive to oxidative stress^36–38^. This suggests a potential role for the impact of oxidative stress on the DDAH1-ADMA axis and resulting changes in intrahepatic vascular tone in cirrhosis patients.

This study aimed to study the bile duct ligated rat model of cirrhosis with PH and inflammation, to determine the effects of IFX therapy on severity of PH, and its role in modulating oxidative-stress and the DDAH1-ADMA axis.

## Materials and Methods

### Animals

Male Sprague Dawley rats (Charles River, UK) were housed in a temperature and light controlled (12 hours light/dark cycle) facility at the Comparative Biology Unit, UCL. Rats received standard chow and water ad libitum. All procedures were performed in accordance with UK Home Office Animals (Scientific Procedures) Act 1986 (updated 2012). The study was approved by the University College London Animal Welfare and Ethical Review Board (AWERB) and a project licence was provided by the UK Home Office.

### Experimental Procedures

Male Sprague Dawley rats weighing 220 ± 25 g underwent sham or BDL surgery as described previously^39^. TNF blocking was performed using Infliximab (IFX; MSD, Hoddesdon, UK). Treatment with either IFX or saline (control, equal volume) by intraperitoneal (i.p.) injection for 3 consecutive days commenced on day 29 post-surgery. 24 hours after the last dose (ie on day 32 following surgery), hemodynamic measurements were performed and samples collected for analysis. Rats were divided into the following treatment groups:

(i) Sham group: sham surgery, i.p. saline (n = 10).
(ii) BDL group: BDL surgery, i.p. saline (n = 10).
(iii) BDL + IFX group: BDL surgery, i.p. IFX 10 mg/kg (n = 7).

### Haemodynamic measurements

Portal pressure and systemic haemodynamics were measured under anaesthesia (2% isofluorane in oxygen). Portal pressure (PP) was measured by direct cannulation of the main portal vein, and mean arterial pressure (MAP) by cannulation of the right carotid artery. All measurements were transduced to a Powerlab (4SP) linked to a computer with Chart v5.0.1 software. The mean of three readings taken one minute apart was recorded. All rats were then sacrificed by exsanguination; blood was withdrawn into heparin or EDTA vacutainers (BD, Oxford, UK), centrifuged (3500 rpm, 10 min, 4 °C) and plasma stored at −80 °C. Liver tissue was snap frozen in Iiquid nitrogen.

### Plasma biochemistry

Plasma ALT and AST activity; and total bilirubin, albumin, and ammonia concentration were measured using a Cobas Integra 400 automated analyser (Roche Diagnostics, Burgess Hill, UK) using the relevant kits according to the manufacturers instructions.

### Hepatic TNF measurement

Liver samples (100 mg) were homogenized in ice-cold Tris-HCl lysis buffer, and protein quantification was performed using the biuret method. TNF cytokine levels were measured in plasma and tissue homogenates using a rat cytometric bead array (CBA) kit (BD, Oxford, UK), according to manufacturer’s instructions. Fluorescence produced by the CBA beads was measured on a FACS Canto II, and data analysed with BD CTA software (BD, Oxford, UK).

### Radiometric analysis of hepatic eNOS activity

The conversion of ^14^C l-arginine to ^14^C l-citrulline was used as a measure of nitric oxide synthase (NOS) activity. A previously determined method was applied with slight modification^40^. Briefly, snap frozen liver tissue was homogenised in Tris-HCl buffer and the protein concentration determined. For NOS activity determination, 5 µl of lysate was incubated with 40 µl of reaction medium [50 mM Tris-HClHCl, pH7.4; 1.25 mM NADPH; 10 µCi/ml ^14^C-arginine (Amersham Biosciences (GE), Little Chalfont, UK); 5 mM norvaline; and either 400 µM CaCl_2_ or 600 µM EGTA] at 30 °C for 30 minutes, and the reaction stopped with 500 µl of ice-cold 50 mM citrate buffer, pH5 containing 1 mM EDTA. The arginine:citrulline ratio was determined by separating the amino acid components using thin layer chromatography on silica plates (Kieselgel 60; Merck, Darmstadt, Germany). Non-tritiated amino acids were added to aid spot detection, and the components separated using a running mixture of CHCl_3_:MeOH:NH_4_OH:H_2_O (10:45:20:10). The individual spots were removed and scintillation activity measured as counts/mg protein/30 min. Reactions performed with CaCl_2_ gave measured total NOS activity. Reactions performed with EGTA (to chelate all calcium) measured iNOS (calcium independent) activity. The difference in measured NOS activity with and without EGTA calcium chelation is interpreted as representing eNOS (calcium dependent) activity.

### Mass spectrometry analysis of ADMA and arginine

Plasma and liver levels of ADMA and arginine were measured using stable isotope dilution Liquid Chromatography-tandem Mass Spectrometry (HPLC-MS; Dionex HPLC system together with Thermo Scientific Orbitrap XL system and Electrospray source). In brief, standards and samples containing ^13^C-ADMA and ^13^C-arginine as internal standards (Cambridge Isotopes, Boston MA, USA) were deproteinised with ice-cold acetonitrile (ACN; 4:1 ACN:sample) and centrifuged at 13,000 rpm at 4 °C for 30 min to remove bulk proteins. Samples were chromatographed (ACN 5–90% water gradient, with 0.1% formic acid) on a 1.9 µm, 50 × 2.1 mm, Pinnacle column (Thames Restek UK Ltd, Saunderton, UK). Analysis was performed on an Orbitrap XL system (ThermoFisher Scientific, Hemel Hempstead, UK) in positive ion mode, using collision induced dissociation (CID). Acquisition time was 6 minutes. Analysis was conducted using Xcalibur software version 2.0.7 (ThermoFisher Scientific, Hemel Hempstead, UK). Plasma concentrations are µmol/L; liver concentrations are µmol/g protein.

### Western blot analysis

Proteins were isolated from snap frozen liver tissue by homogenising in ice-cold Tris-HCl (50 mM, pH 7.6) with protease inhibitor cocktail (Sigma-Aldrich Company Ltd., Gillingham, UK), PMSF, and EDTA. The protein concentration was determined using Bradford reagent. Cultured cells were lysed in ice-cold RIPA buffer. The protein concentration was measured using a BCA assay kit (Life Technologies Ltd., Paisley, UK). Equal amounts of protein were electrophoresed through 4–12% NuPAGE Bis-Tris Gels and transferred to PVDF membranes (Life Technologies Ltd., Paisley, UK). Blots were probed with the following primary antibodies: rabbit anti-DDAH1 (Abcam, Cambridge, UK); mouse anti-eNOS (BD, Oxford, UK), mouse anti-NF-κB, (Cell Signalling Technology, Danvers, MA, USA), mouse anti-4HNE (Abcam, Cambridge, UK), rabbit anti-Vegf-A (Santa Cruz Biotechnology, Inc, Dallas, TX, USA), mouse anti-Gapdh (loading control; Abcam, Cambridge, UK), and mouse anti-α-tubulin (loading control; Millipore, Watford, UK). Immune complexes were detected using HRP-conjugated secondary antibodies (Cell Signalling Technology, Danvers, MA, USA) and enhanced chemiluminescence (ECL) reagents (GE Healthcare Lifesciences, Little Chalfont, UK). Densitometric quantification was performed using Image J (US NIH, Bethesda, MD, USA; http://imagej.nih.gov/ij/).

### Cell culture

HepG2 cells were cultured in DMEM with 10% FBS (Life Technologies Ltd., Paisley, UK) at 37 °C in a 5% CO_2_ atmosphere. HepG2 cells were exposed to either 10 uM H_2_O_2_, 100 uM H_2_O_2_, or control media for 24 hours. All measurements were performed in duplicate.

### Luciferase reporter assays

The 3′ UTR of human DDAH1 was amplified from human cDNA using the PCR primers F: CGTGAGCATGTCTGAACTGG and R: CATGATTGGTTTTGGCACAC. The PCR product was TA-cloned using the pGEM-T Easy kit (Promega, Southampton, UK), followed by restriction enzyme subcloning into the pMirReport luciferase reporter vector (Life Technologies Ltd., Paisley, UK) to produce pMR-DDAH1. HepG2 cells were co-transfected with 0.5ug pMR-DDAH1 and 0.1ug pRL-CMV (expressing Renilla Luciferase as transfection control; Promega, Southampton, UK) using Fugene 6 (Roche Diagnostics, Burgess Hill, UK) in a reverse transfection protocol according to the manufacturer’s recommendations. 16 hours following transfection cells were exposed to 0, 10, or 100 uM H_2_O_2_ for 24 hours. Cells were lysed and firefly and Renilla luciferase activity measured using the Dual-Luciferase Reporter assay (Promega, Southampton, UK). Data is expressed as firefly/Renilla luciferase activity. All measurements were performed in triplicate.

### Statistics

Data are presented as mean and standard error. T-test, Mann Whitney U test, and one-way annova were performed using GraphPad Prism (version 5.03 for Windows; GraphPad Software, San Diego, CA, USA).

### Data availability

All data generated or analysed during this study are included in this published article and its Supplementary Information files.

## Results

### TNF blockade with IFX reduces portal pressure in cirrhotic rats with portal hypertension

The bile duct ligation (BDL) rat model was used as it replicates advanced cirrhosis in patients, demonstrating portal hypertension and extra-hepatic organ failure^41^. All rats were studied 28-days following BDL or sham surgery. All animals continued to gain weight following surgery. BDL rats gained less weight than sham rats leading to a lower body weight at termination (Table 1). IFX treatment led to an increase in body weight at termination (Table 1).

**Table 1:** Physical and haemodynamic measurements.

	Sham	BDL	BDL + IFX
Body weight at termination (g)	476.6 (10.3)	332 (6.2)****	357 (8.2)^#^
Portal pressure (mmHg)	6.30 (0.212)	14.50 (0.827)****	9.48 (0.697)^##^
MAP (mmHg)	122.5 (2.06)	85 (3.99)**	83.9 (0.92)

Data are shown as mean (standard error). **p < 0.01, ****p < 0.0001 compared to Sham. ^#^p < 0.05, ^##^p < 0.01 compared to BDL. (Sham n = 10; BDL n = 10; BDL + IFX n = 7).

**Figure 1:**
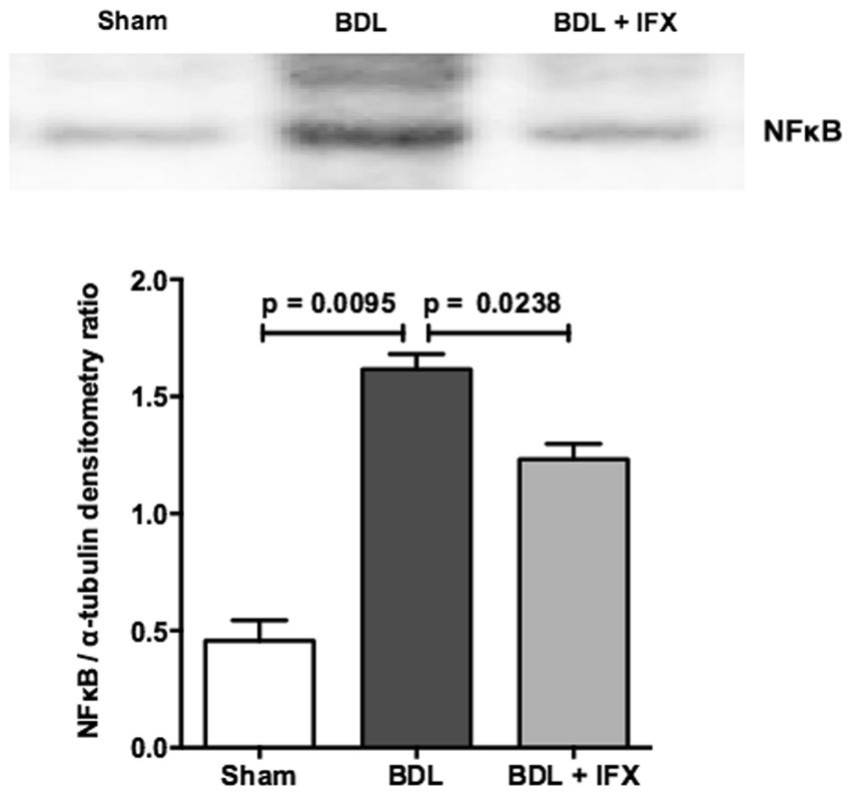
NFkB expression in rat liver. Western blotting shows that expression of the p65 subunit of NFkB is increased in BDL rat livers compared to sham, and is decreased by IFX treatment. (Sham n = 4; BDL n = 6; BDL + IFX n = 3).

To confirm that the IFX treatment was successful, we measured the liver and plasma levels of TNF and the liver expression of NFkB, a downstream effector of TNF signalling. BDL rats had increased liver and plasma levels of TNF compared to sham rats and this was reduced by IFX (Table 2). NFkB was also increased in BDL liver compared to Sham, and was reduced by IFX treatment (Fig. 1).

**Table 2:** Plasma and liver TNF levels and plasma biochemistry measurements.

	Sham	BDL	BDL + IFX
Plasma TNF (pg/ml)	121.9 (46.4)	822.9 (203.1)****	140.4 (36.7)^#^
Liver TNF (pg/mg protein)	0.3 (0.03)	0.51 (0.03)**	0.42 (0.01)^#^
ALT (U/L)	35.43 (3.94)	122.8 (17.4)****	72.08 (7.22)^#^
AST (U/L)	82.24 (3.31)	626.7 (127.6)****	426.7 (97.6)^#^
Bilirubin (μmol/L)	35.14 (4.02)	202.0 (18.62)****	203.0 (20.22)
Ammonia (μmol/L)	56.68 (5.89)	170.6 (14.87)**	120.00 (15.44)^#^
Albumin (g/L)	33.03 (0.5)	18.06 (0.75)****	24.13 (1.28)^#^

Data are shown as mean (standard error). **p < 0.01, ****p < 0.0001 compared to Sham. ^#^p < 0.05 compared to BDL. (Sham n = 10; BDL n = 10; BDL + IFX n = 7).

To determine whether IFX treatment of BDL rats can recapitulate the reduction in portal pressure previously demonstrated in patients^15^, we measured haemodynamic parameters. BDL rats had significantly elevated portal pressure compare to Sham (Table 1; 14.50 ± 0.83 mmHg vs 6.30 ± 0.21 mmHg; p < 0.0001). BDL rats treated with IFX had significantly reduced portal pressure compared to BDL alone (Table 1; 9.48 ± 0.70 mmHg vs 14.50 ± 0.83 mmHg; p < 0.01), confirming the BDL rat model is suitable for studying the mechanistic effects of IFX on portal hypertension. BDL rats had significantly lower mean arterial pressure (MAP) compared to sham rats and this was not altered by IFX treatment (Table 1) indicating that the reduction in portal pressure post IFX therapy was not due to an overall reduction in liver perfusion pressure.

### IFX treatment reduces markers of liver injury, inflammation and oxidative stress in BDL rats

Plasma biochemistry analysis of common markers of liver injury and inflammation showed that BDL rats had significantly elevated ALT, AST, bilirubin, and ammonia, compared to Sham rats (Table 2). They also had significantly reduced Albumin (Table 2). Treatment with IFX significantly decreased ALT, AST, and ammonia, and significantly increased albumin (Table 2).

**Figure 2:**
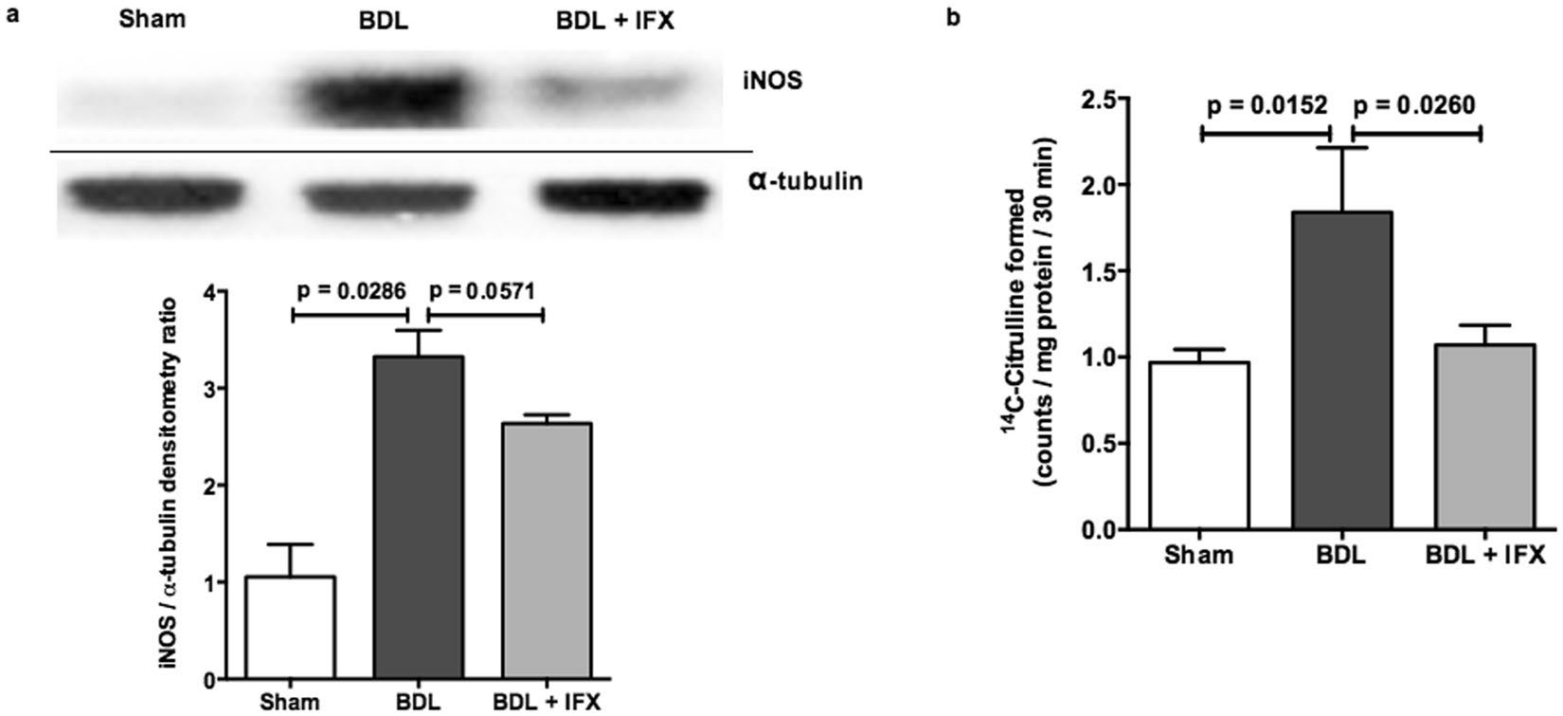
iNOS expression and activity in rat liver. (**a**)Western blot analysis shows increased hepatic iNOS protein expression in BDL rat livers compared to sham, and this is decreased in BDL rats treated with IFX. Following detection of iNOS, western blots were stripped and reprobed for detection of α-tubulin. (Sham n = 4; BDL n = 4; BDL + IFX n = 4). (**b**) iNOS activity is increased in livers of BDL rats compared to sham. Treatment with IFX reduces iNOS activity in BDL rat livers. (Sham n = 6; BDL n = 6; BDL + IFX n = 6).

The activity and expression of hepatic iNOS (a mediator of inflammation), were measured by a radiometric assay and Western blotting respectively. Both iNOS activity and protein expression were increased in BDL rat liver compared to sham, and were decreased by IFX treatment (Fig. 2).

To determine the level of oxidative stress in the rat livers, the formation of 4-HNE protein adducts (a marker of lipid peroxidation) were studied by Western blotting. Densitometric analysis of the predominant band showed an increase in 4-HNE adducts in BDL rats compared to sham, and this was reduced by IFX treatment (Supplementary Fig. S1).

### TNF blockade improves hepatic eNOS activity in cirrhotic rats

To determine whether a reduction in fibrosis could be involved in the reduction in portal pressure we performed Western blot analysis of the fibrotic marker Acta2. Acta2 was increased in BDL rat livers compared to sham, and this was not reduced or altered by treatment with IFX (Supplementary Fig. S2), indicating that short-term IFX treatment does not reduce liver fibrosis. In addition, H&E staining showed that short-term IFX treatment was not able to reverse the architectural disturbances found in BDL liver (data not shown). IFX treatment reduced Vegf-A protein expression in rat liver (Supplementary Fig. S3), suggesting a reduction in hepatic stellate cell activity.

**Figure 3:**
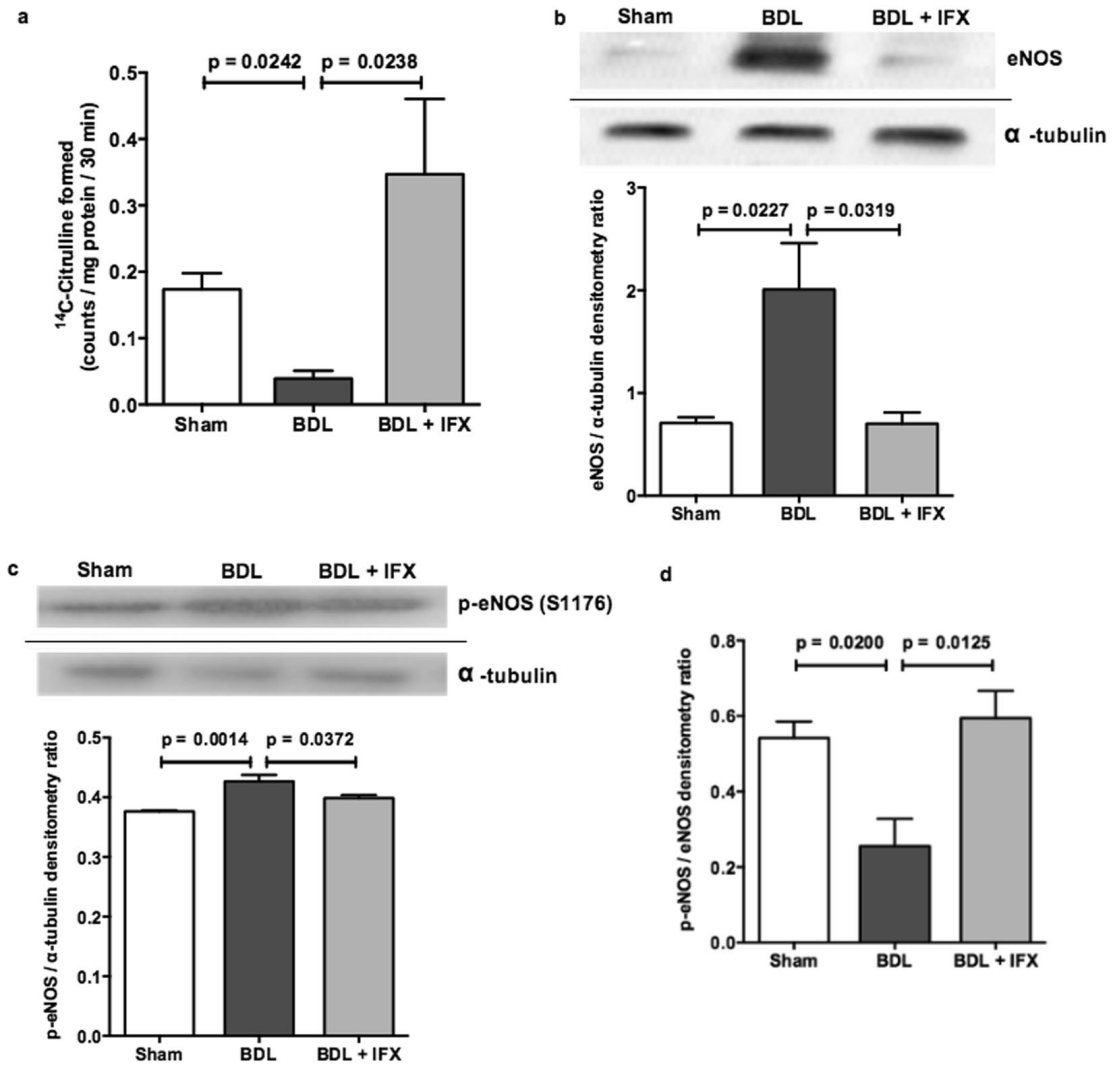
eNOS expression and activity in rat liver. (**a**) eNOS activity is decreased in BDL rat liver compared to sham, and is increased by treating BDL rats with IFX. (Sham n = 7; BDL n = 4; BDL + IFX n = 3). (**b**)Western blot analysis shows that eNOS protein expression is increased in BDL rat liver compared to sham, and is reduced by treating BDL rats with IFX. Following detection of eNOS, western blots were stripped and reprobed for detection of α-tubulin. (Sham n = 4; BDL n = 4; BDL + IFX n = 3). (**c**)Western blot analysis shows that the phosphorylated eNOS (p-eNOS, ser-1177) active form is increased in BDL rat liver compared to sham, and is reduced by treating BDL rats with IFX. Following detection of p-eNOS, western blots were stripped and reprobed for detection of α-tubulin. (Sham n = 4; BDL n = 4; BDL + IFX n = 3). (**d**)The ratio of p-eNOS to eNOS densitometry (from panels b and c) is reduced in BDL rat liver compared to sham, and is increased with IFX treatment. (Sham n = 4; BDL n = 4; BDL + IFX n = 3).

To determine whether a change in NO levels could be involved in the reduction in portal pressure, the activity of eNOS in the rat livers was measured by a radiometric assay, and the protein expression levels were determined by Western blotting. Hepatic eNOS activity was significantly lower in BDL rats compared to sham (Fig. 3a). Treatment of BDL rats with IFX caused a significant increase in eNOS activity (Fig. 3a). Despite this, hepatic expression of eNOS protein was significantly increased in BDL rats compared to sham, and was significantly reduced by IFX treatment (Fig. 3b). In addition, the phosphorylated eNOS (p-eNOS) active form was also increased in BDL rats compared to sham, and was reduced by IFX treatment (Fig. 3c). The ratio of p-eNOS/eNOS was significantly reduced in BDL rats compared to sham, and significantly increased by IFX treatment (Fig. 3d).

### TNF blockade leads to an improved l-arginine/ADMA ratio and increased hepatic DDAH1 expression in cirrhotic rats

The reduction in eNOS activity despite an increase in its expression suggested the presence of eNOS inhibitors. Therefore, we measured the levels of the eNOS substrate, l-arginine, and the endogenous eNOS inhibitor, ADMA, in the plasma and liver. ADMA was increased in BDL rat liver compared to Sham, and was reduced by IFX treatment (Table 3). Similarly, plasma ADMA was increased in BDL rats compared to sham, and reduced by IFX treatment (Table 3). The ratio of l-arginine/ADMA was reduced in both liver and plasma of BDL rats compared to sham, and was improved by IFX treatment (Table 3).

**Table 3:** Liver and plasma l-arginine and ADMA measurements.

	Sham	BDL	BDL + IFX
Liver ADMA (μmol/g protein)	0.16 (0.01)	2.43 (0.207)****	0.25 (0.029)^####^
Plasma ADMA (μmol/L)	0.51 (0.010)	1.03 (0.093)****	0.79 (0.154)^#^
Liver l-arginine/ADMA ratio	1.62 (0.157)	0.85 (0.067)*	5.85 (0.358)^##^
Plasma l-arginine/ADMA ratio	257 (20.26)	81.79 (25.91)*	162.8 (38.47)^#^

Data are shown as mean (standard error). *p < 0.05, ****p < 0.0001 compared to Sham. ^#^p < 0.05, ^##^p < 0.01^, ####^p < 0.0001 compared to BDL. (Sham n = 10; BDL n = 10; BDL + IFX n = 7).

**Figure 4:**
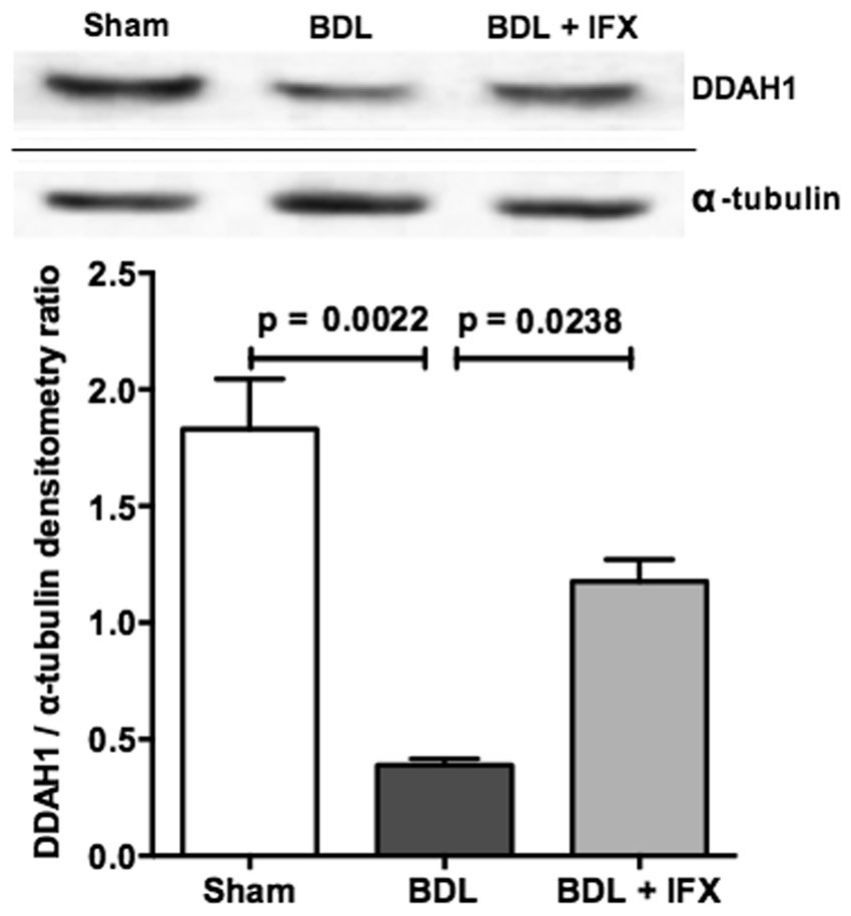
DDAH1 expression in rat liver. Western blot analysis shows that DDAH1 protein expression is reduced in livers of BDL rats compared to sham rats, and is increased in BDL rats treated with IFX. Following detection of DDAH1, western blots were stripped and reprobed for detection of α-tubulin. (Sham n = 6; BDL n = 6; BDL + IFX n = 3).

We performed Western blot analysis to determine whether the changes in ADMA concentrations could be explained by the levels of its main catabolic enzyme, DDAH1. As shown in Fig. 4, expression of DDAH1 was reduced in BDL rat livers compared to sham, and was increased by IFX treatment. In contrast, DDAH2 protein expression was increased in BDL liver compared to sham and was unchanged by IFX treatment (Supplementary Fig. S4).

### DDAH1 expression is reduced by oxidative stress through a post-transcriptional mechanism

**Figure 5:**
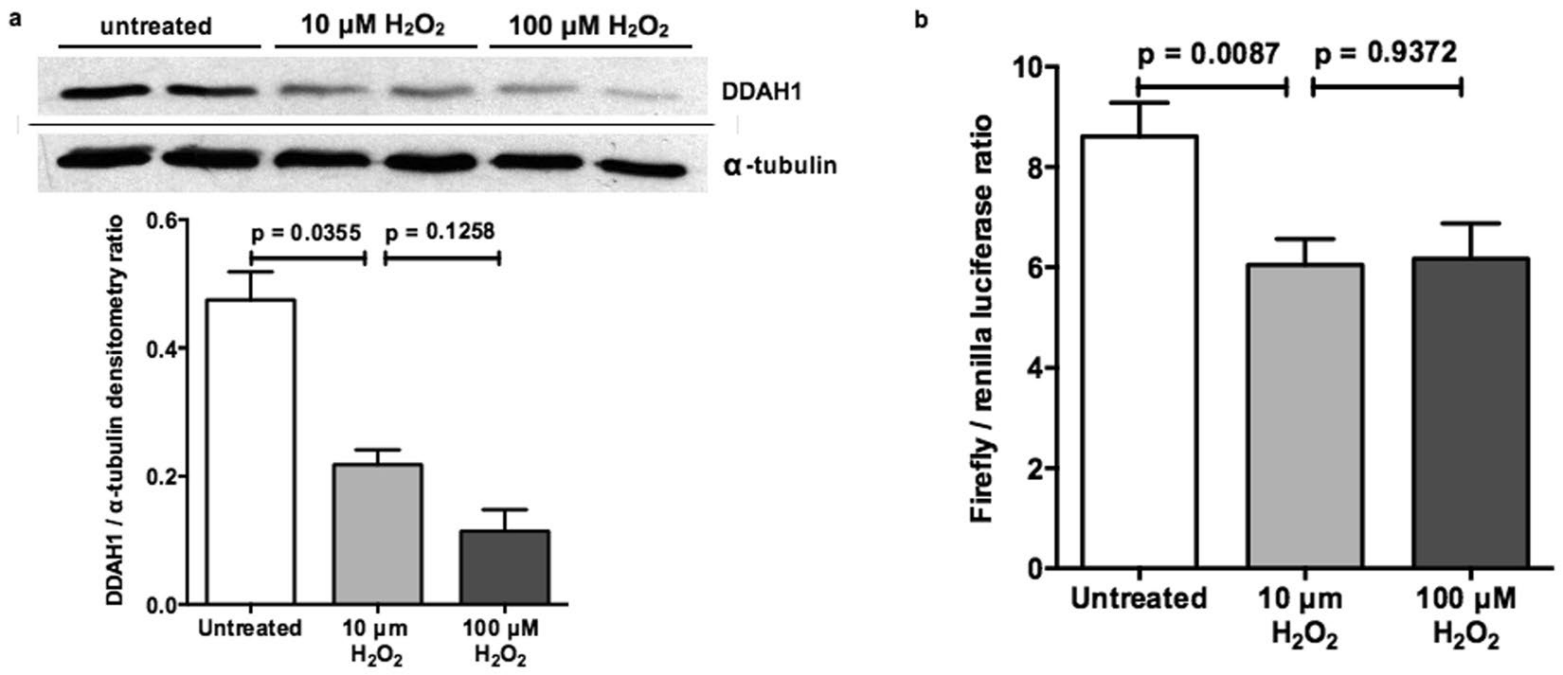
Oxidative stress reduces DDAH1 expression in hepatocytes by a post-transcriptional mechanism. (**a**)Western blot analysis of HepG2 cells treated with 10 uM H2O2 show reduced DDAH1 protein expression compared to untreated cells. DDAH1 protein is further reduced in cells treated with 100 uM H2O2. Western blots were probed for DDAH1 and α-tubulin simultaneously. (Untreated n = 2; 10 uM n = 2; 100 uM n = 2).(**b**) HepG2 cells were transfected with a plasmid with the DDAH1 3′UTR cloned downstream of the firefly luciferase coding sequence and luciferase activity (normalised to co-transfected Renilla luciferase activity) was measured. Cells treated with either 10 uM or 100 uM H2O2 showed reduced luciferase activity compared to untreated cells. (Untreated n = 6; 10 uM n = 6; 100 uM n = 6).

Having shown that IFX decreases markers of oxidative stress and increases eNOS activity and DDAH1 expression, we sought to determine if there was a link between oxidative stress and DDAH1 expression. The effect of H_2_O_2_-induced oxidative stress on cultured hepatocytes was studied. DDAH1 expression was measured in HepG2 cells exposed to 0, 10, and 100 uM H_2_O_2_ by Western blot. DDAH1 protein expression was reduced by 10 uM H_2_O_2_ and was further reduced by 100 uM H_2_O_2_ (Fig. 5a). To determine whether miRNA regulation may play a role in DDAH1 expression, we analysed the DDAH1 3′UTR activity following induction of oxidative stress by H_2_O_2_. HepG2 cells were transfected with a plasmid containing the 3′UTR of human DDAH1 cloned downstream of the firefly luciferase coding sequence (pMR-DDAH1). The luciferase activity was measured following treatment of the cells with 0, 10, or 100 uM H_2_O_2_. As shown in Fig. 5b, both 10 and 100 uM H_2_O_2_ reduced luciferase activity indicating an increased regulatory action of the DDAH1 3′UTR following oxidative stress.

## Discussion

This study adopted *in vitro* and *in vivo* experimental approaches in an attempt to provide mechanistic information for our prior clinical observation of a significant reduction in portal pressure following IFX therapy, in cirrhosis patients with acute alcoholic hepatitis induced decompensation^15^. The focus of the study was to assess the impact of acute reduction in inflammation with an anti-TNF strategy upon pathways that modulate hepatic vascular tone, whilst preserving mean arterial pressure, in environments of inflammation and oxidative stress. The key findings from this study were: (i) BDL cirrhotic rats demonstrate elevated TNF and NFkB signalling, and their reduction by anti-TNF therapy is associated with a lowering of portal pressure; (ii) TNF blockade leads to decreased hepatic oxidative stress, and reduced hepatic inflammation and biochemical injury; (iii) lowering portal pressure with an anti-TNF intervention is associated with an improvement in endothelial NOS activity, an increase in the liver arginine/ADMA ratio, and increased DDAH1 expression; (iv) hepatocyte DDAH1 expression *in vitro* is decreased by oxidative stress, and this reduction in DDAH1 expression is partially mediated by altered post-transcriptional regulation.

Several lines of evidence implicate systemic and hepatic inflammation as key to the pathobiology of portal hypertension in advanced cirrhosis^42,43^. Linear correlations have been demonstrated between markers of systemic inflammation, such as serum CRP, IL-6 and IL-1β levels, and hepatic venous pressure gradient (HVPG)^44–46^. Moreover, Kupffer cell (KC) activation, as measured by plasma sCD163 levels, correlate with HVPG and incidence of variceal hemorrhage^47,48^. This suggests that gut bacterial translocation may drive KC activation and, together with drivers for liver injury such as alcohol, produce hepatic inflammation in cirrhosis decompensation. Translocation of bacteria, or bacterial products, leads to the activation of toll-like receptors (TLRs) primarily on KCs leading them to adopt a pro-inflammatory phenotype, shedding sCD163 and producing pro-inflammatory cytokines such as TNF, IL-1 and IL-6^49^. This pro-inflammatory response is in turn associated with severe portal hypertension^50,51^. Serum bacterial DNA levels, as a surrogate marker of translocation, are correlated with severity of inflammation and portal hypertension in cirrhosis^3^. Additionally, in patients with spontaneous bacterial peritonitis, elevated levels of TNF are associated with higher HVPG^4^.

The role of NO as a regulator of intrahepatic vascular tone has been known for three decades, and decreased intrahepatic bioavailability of NO is recognized to be a key cause of sinusoidal endothelial dysfunction in portal hypertension^23^. However, as re-confirmed in our study, this occurs despite increased hepatic eNOS protein expression in cirrhosis^27,52,53^. Both post-translational modifications of eNOS and the presence of eNOS inhibitors have been shown to be valid mechanisms to account for decreased intrahepatic NO generation^32,54,55^. Of these, ADMA is emerging as an important endogenous inhibitor of eNOS and has been identified as the main determinant of endothelial dysfunction in conditions such as reno-vascular disease and pulmonary hypertension^56,57^. Plasma ADMA levels are also markedly increased in the plasma of patients with liver disease, and correlate with severity of portal hypertension, onset of organ dysfunction and outcome^32,58^. The main route of elimination of ADMA is catabolism, predominantly by the enzyme DDAH1, which is primarily expressed in the liver and kidney^59^. Our group previously demonstrated that hepatic expression of DDAH1 is restricted to the hepatocyte rather than non-parenchymal cells, and that hepatic expression of DDAH1 is decreased in BDL cirrhotic rats^35^. Moreover, DDAH1 has been shown to be sensitive to TNF-mediated inflammation and oxidative stress. DDAH1 activity is decreased in endothelial cells exposed to TNF or oxidized LDL^60^. Renal preglomerular vascular smooth muscle cells (VSMCs) exposed to oxidative stress with H_2_O_2_ showed a marked decrease in DDAH1 protein expression^61^.

In the current study, we confirmed our previous finding of decreased hepatic eNOS activity in association with reduced hepatic DDAH1 protein expression in BDL cirrhotic rats, and also validated that this model is characterised by hepatic inflammation and oxidative stress. As a pro-inflammatory cytokine, TNF activates the NF-κB pathway leading to a downstream cascade of other pro-inflammatory cytokines as well as mitochondrial reactive oxygen species (ROS) generation, inducing the formation of lipid-derived 4-HNE. Thus, as expected, TNF blockade in BDL cirrhotic rats led to decreased hepatic TNF, decreased NF-κB activation, and decreased hepatic lipid peroxidation. However, the mechanism for this association between ROS formation and sinusoidal endothelial dysfunction remains an area of active investigation. Increased generation of superoxide has been demonstrated in cirrhotic liver, and proposed to lead to scavenging for NO, hence contributing to decreased NO bioavailability alongside decreased eNOS activity^62^. In this study we demonstrate a further mechanism for hepatic ROS generation and sinusoidal endothelial dysfunction through decreased hepatocyte DDAH1 expression, leading to increased hepatic ADMA and eNOS dysfunction. Anti-TNF therapy led to a physiologically relevant reduction in hepatic ADMA such that the ratio of hepatic l-arginine/ADMA was significantly increased leading to improved eNOS activity.

A novel finding in this study was the demonstration that hepatocyte DDAH1 is sensitive to oxidative stress, with a concentration-dependent decrease in DDAH1 protein expression. Importantly, these findings echo the findings of Luo *et al*. who found that exposure of VSMCs to H_2_O_2_ led to decreased DDAH1 protein expression but increased DDAH1 mRNA expression^61^. These authors found that proteasomal inhibition led to partial but not complete reversal of this effect, suggesting that the decrease in DDAH1 protein expression is partially due to protein degradation but the remainder of this effect remained unexplained. In the current study we proceeded to evaluate the 3′UTR of DDAH1, since post-transcriptional mRNA interactions in the 3′UTR have been shown to account for decreased protein expression in response to H_2_O_2_-mediated oxidative stress in other cell types^63^. Our findings demonstrate that the DDAH1 3′UTR is regulatory in hepatocytes, and 10 uM concentrations of H_2_O_2_ lead to a repression of luciferase expression which is not enhanced at the higher 100 uM concentration. Thus, it is likely that post-transcriptional regulation of DDAH1 mRNA is partially responsible for the decrease in DDAH1 protein expression seen in hepatocytes during times of oxidative stress, leading to increased ADMA and consequent endothelial dysfunction.

The results of this study could also be interpreted as TNF blockade having a direct effect on eNOS activity, through decreasing ROS-induced eNOS uncoupling^54^. However, our results indicate a robust increase in hepatic and plasma ADMA in cirrhotic rats, which is ameliorated by anti-TNF, suggesting that augmentation of the DDAH-ADMA axis is responsible for at least part of the improvement in eNOS activity observed. In concert with observations by other investigators, this study supports an emerging role for hepatic and systemic inflammation in the pathobiology of portal hypertension in acute decompensation of cirrhosis. The mechanisms are likely to be complex, and involve cross-talk between Kuppfer cells, stellate cells and endothelial cells in the sinusoidal niche. In the context of inflammation and oxidative stress, the production of ADMA by hepatocytes as a consequence of decreased DDAH1 activity is likely to act as a paracrine mediator causing eNOS dysfunction in the sinusoidal endothelium. Although a paracrine role for ADMA signalling between parenchymal cells and endothelial cells has been proposed in the kidney^61^, further work will be required to confirm this hypothesis in the liver. Since anti-TNF therapy has not been translated to patients with decompensated cirrhosis due to the high risk of bacterial infection, future work will also determine whether alternative strategies to modulate oxidative stress or the post-transcriptional regulation of DDAH1 in environments of oxidative stress are of benefit in treating portal hypertension.

## Electronic supplementary material

Supplementary figures
