# Supplementary material for: Post-Transcriptional Regulation of Hepatic DDAH1 with TNF Blockade Leads to Improved eNOS Function and Reduced Portal Pressure In Cirrhotic Rats
Source: Sci Rep. 2017 Dec 20;7:17900. doi: 10.1038/s41598-017-18094-3 (PMC5738445; doi:10.1038/s41598-017-18094-3)
Supplement: Supplementary file 1 — Supplementary figures [file 41598_2017_18094_MOESM1_ESM.pdf]

Post-Transcriptional Regulation of Hepatic DDAH-1 with TNF Blockade Leads to Improved eNOS Function and Reduced Portal Pressure In Cirrhotic Rats

V Balasubramanian, G Mehta*, H Jones, V Sharma, N Davies, R Jalan, RP Mookerjee*

** These authors contributed equally*

Supplementary Figures

Supplementary Figure S1

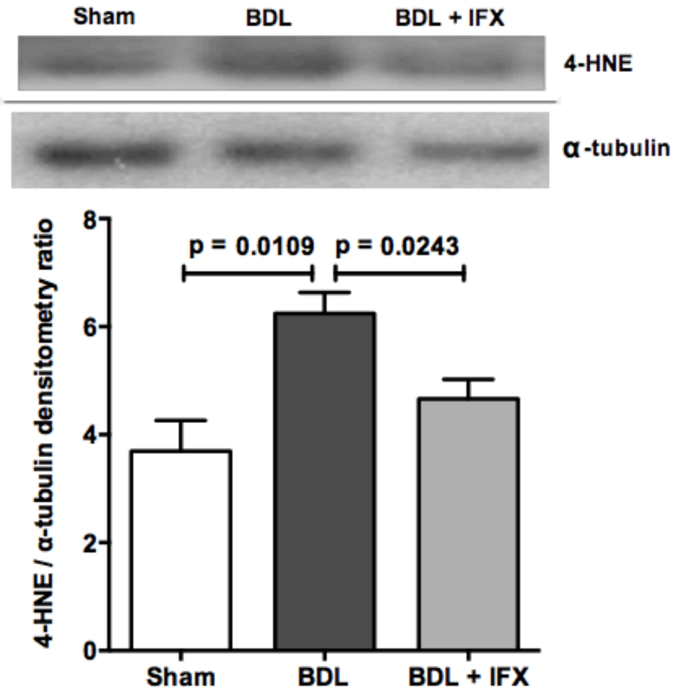

Lipid peroxidation in rat livers

Lipid peroxidation was assessed by measuring 4-HNE protein adducts in rat livers by western blotting. Densitometry of the predominant band observed was normalised to densitometry of α-tubulin. 4-HNE protein adducts were increased in livers of BDL rats compared to sham, and this was decreased in rats treated with IFX. Following detection of 4-HNE, western blots were stripped and reprobed for detection of α-tubulin. (Sham n=6; BDL n=4; BDL + IFX n=4).

Supplementary Figure S2

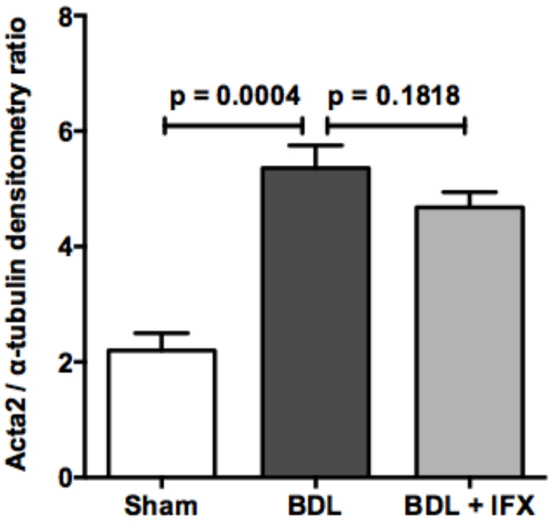

Acta2 expression in rat liver

Acta2 protein expression was analysed by western blot. Densitometry of Acta2 was normalised to densitometry of α-tubulin. Acta2 protein expression is increased in BDL rat liver compared to sham. It is not reduced by short-term treatment with IFX. Following detection of Acta2, western blots were stripped and reprobed for detection of α-tubulin. (Sham n=4; BDL n=6; BDL + IFX n=6).

Supplementary Figure S3

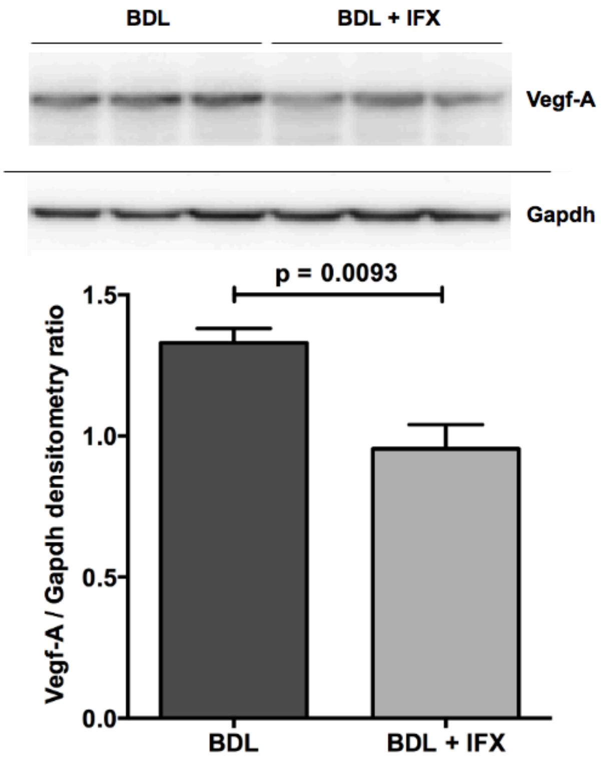

Vegf-A expression in rat liver

Vegf-A protein expression was analysed by western blot. Densitometry of Vegf-A was normalised to densitometry of Gapdh. Vegf-A protein expression is reduced by treatment with IFX. Following detection of Vegf-A, western blots were stripped and reprobed for detection of Gapdh. (BDL, n=4; BDL + IFX, n=4).

Supplementary Figure S4

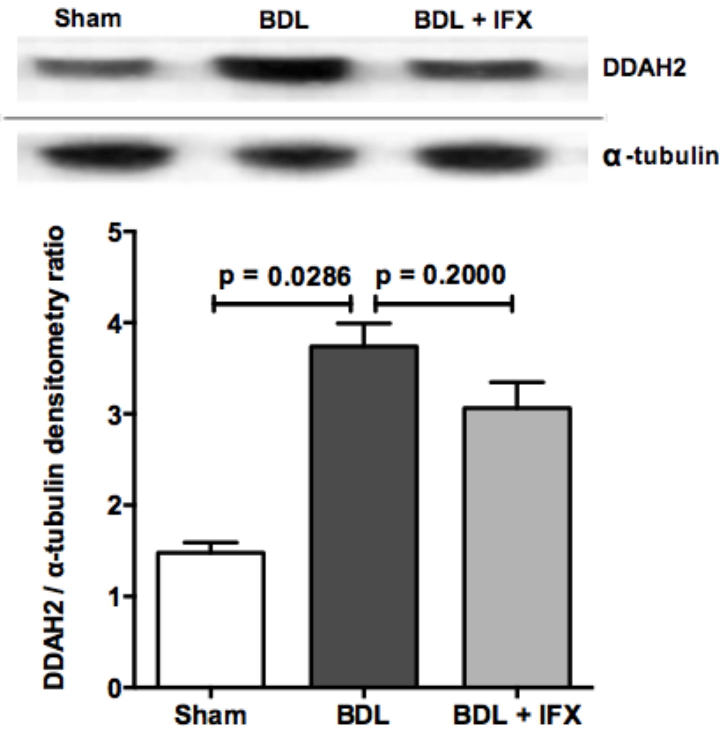

DDAH2 expression in rat liver

DDAH2 protein expression was analysed by western blot. DDAH2 densitometry was normalised to α-tubulin densitometry. DDAH2 expression is increased in livers of BDL rats compared to sham, but is not decreased by treatment with IFX. Following detection of DDAH2, western blots were stripped and reprobed for detection of α-tubulin. (Sham n=4; BDL n=4; BDL + IFX n=4)
